# Plasma-Induced Tailoring of Graphene Oxide Surfaces for Electrochemical Applications: Functionalization and Etching

**DOI:** 10.1021/acsaelm.5c00939

**Published:** 2025-06-29

**Authors:** Yijing Y. Stehle, Timothy J. Barnum, Sandra Schujman, Ivan V. Vlassiouk, Rebecca Cortez

**Affiliations:** † Department of Mechanical Engineering, 7254 Union College, Schenectady, New York 12308, United States; ‡ Department of Chemistry, Union College, Schenectady, New York 12308, United States; § NY CREATES, Albany, New York 12203, United States; ∥ Center for Nanophase Materials Sciences, 6146 Oak Ridge National Laboratory, Oak Ridge, Tennessee 37831, United States

**Keywords:** graphene oxide (GO), radio frequency air plasma, superhydrophilic, surface modification, etching

## Abstract

This study investigates the use of radio frequency air plasma as an eco-friendly method to rapidly and reversibly tailor the surface properties of graphene oxide (GO) films. We observed a transition from hydrophilic (contact angle ∼55°) to superhydrophilic (<10°) with short plasma exposure, attributed to a synergistic combination of surface modification and etching. Spectroscopic analyses (FTIR, XPS) revealed early stage formation of carbonyl groups and reduction of hydroxyls, while longer treatments induced atomic-level etching (AFM) and structural changes (XRD). This surface engineering enhanced the dielectric properties of GO films but led to reduced aqueous stability. The elucidated interplay between plasma-induced functionalization and etching provides valuable insights for the controlled modification of GO surfaces for various applications, including advanced dielectrics.

## Graphical abstract

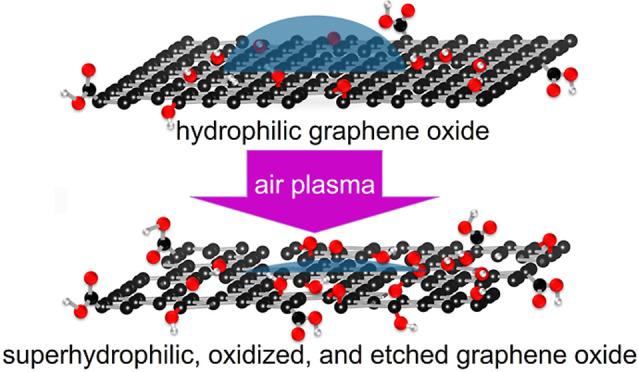

## Introduction

Graphene oxide (GO), a derivative of graphene containing both sp^2^- and sp^3^-hybridized carbon atoms, exhibits a remarkable versatility owing to the presence of oxygenated functional groups (OFGs) such as hydroxyl, epoxy, ketone, and carboxyl groups. These OFGs, decorated on the plane or at the edge of graphene sheets, endow GO with immense potential across various fields, particularly in separation and energy storage applications.^1–3^ The manipulation of these OFGs allows for tailored adjustments in the highly selective permeability and dielectric properties of GO membranes. One of the most effective methods for altering the properties of GO is plasma treatment.^4–9^ Plasma, characterized by ionized gases, has emerged as a rapid, cost-effective, and scalable method for surface modification. The versatility of plasma lies in its ability to introduce defects and functional groups, enhancing or altering material properties based on the treatment conditions.

Previous studies have extensively explored the effects of air plasma treatment on GO, often attributing its impact to collision, oxidation, and exfoliation processes.^10^ For instance, energetic particle bombardment can activate or remove oxygen-containing groups from the GO surface, increasing the atomic C/O ratio and enhancing electrical conductivity. Moreover, the reductive electrons and radicals present in air plasma can induce deoxygenation reactions at the GO surface, further modifying its properties.^10,11^ Additionally, the generation of gaseous products like H_2_O, CO_2_, and O_2_ during plasma treatment can lead to rapid exfoliation and expansion of graphene sheets, with higher plasma power accelerating these processes.^12^

The effectiveness of plasma treatment is not only limited to chemical modifications but extends to physical alterations as well. For example, studies by Flaherty and Qiao have demonstrated that plasma-induced polar functionalization and surface roughness modifications can significantly enhance adhesion between carbon fiber and matrices.^13,14^ Similarly, Vlassiouk et al. employed oxygen plasma etching to enhance the proton conduction and create atomically thin desalination membranes with nanoscale pores,^8,9^ while Zhao improved the sensitivity and stability of humidity sensors based on laser-scribed MoS_2_/GO through low-temperature oxygen plasma treatment.^15^ Furthermore, Losic et al. reported exceptional electrochemical performance in supercapacitors fabricated with plasma-reduced GO.^16–20^ Jeong categorized the effects of plasma treatment on carbon materials into two main outcomes: improving surface polarity, conductivity, and wettability by adding polar groups, and increasing electrochemical surface area through exfoliation.^21–23^

Various reactive gases, including nitrogen, oxygen, hydrogen, and their mixtures, are utilized to tailor plasma-induced defects in graphene, enhancing proton conduction, and provide functionalization.^24–31^ For instance, the hydrogen atmosphere commonly serves as a reduction medium in plasma treatment, albeit it may induce defects due to the shrinkage of C–O bonds.^24^ Conversely, nitrogen atmosphere is employed to mitigate the generation of surface defects during plasma treatment,^25^ while oxygen plasma treatment effectively infuses functional groups, enhancing GO’s electrical conductivity, surface roughness, transparency (>90%), and substrate adhesion.^26,27^ Air plasma, characterized by its swiftness and effectiveness in GO modification, reduction, and exfoliation, operates through physical bombardment and chemical reduction mechanisms.^10,11^ In particular, RF air plasma has emerged as an effective, cost-efficient, safe, and environmentally friendly method for imparting high-quality surface properties and functionalization features to GO films.^6,7^ Despite these advancements, the precise control and comprehensive understanding of plasma effects on GO remain underexplored. Specifically, the coexistence of various reactive species—namely nitrogen, oxygen, and residual water molecules—within an air plasma atmosphere can lead to complex and often conflicting modification pathways on the GO surface, manifesting as both reduction and oxidation effects.^25–27^ The variability in plasma type, energy, atmosphere, and exposure time introduces significant challenges in predicting and tailoring the properties of GO films.

With these objectives in mind, this study introduces a room-temperature RF air plasma strategy for the efficient and eco-friendly surface modification of GO, specifically aimed at enhancing its dielectric properties for improved electrode/dielectric layer interfaces. We experimentally investigate the impact of RF air plasma on the surface modification and etching of GO. A comprehensive set of characterization techniques, including scanning electron microscopy (SEM), X-ray photoelectron spectroscopy (XPS), atomic force microscopy (AFM), X-ray diffraction (XRD), thermogravimetric analysis (TGA), Fourier transform infrared spectroscopy (FTIR), and contact angle (CA) measurements, are employed to analyze the changes in chemical structure, morphology, and crystallinity of GO. Finally, the practical applications of plasma-treated GO films are demonstrated through the fabrication and characterization of a sandwich capacitor configuration, with GO/plasma-treated GO (PGO) films placed between two layers of carbon paper.

## Experimental Section

### Materials and Sample Preparation

Graphene oxide water dispersions (4 mg/mL) were purchased from Graphenea (Cambridge, MA). Free-standing GO membranes were obtained by an ambient evaporation drying process in a Teflon evaporating dish.^32^ The GO coating was prepared by drop casting 2–5 mL of diluted GO solution (0.4 mg/mL) on glass slides and drying under ambient conditions. The individual GO sheets were prepared by diluting the GO solution with deionized water by a factor of 100. The dispersion was further agitated using a probe sonicator for 10 min to obtain a uniform dispersion. It was then drop-coated onto a SiO_2_/Si wafer and dried with compressed air. Air plasma treatments were carried out in a low-power radio frequency plasma cleaner (Harrick Plasma, PDC-32G). The chamber was pumped down to a static working pressure of 200 mTorr, with the plasma generated from the residual ambient air. A consistent plasma power of 18 W was applied, and treatment durations were varied from 20 s to 20 min to investigate their effect on the GO samples. The treatments were conducted at an ambient room temperature of 21 °C and a relative humidity range of 20–30% RH, parameters that were controlled to minimize environmental variability.

### Measurement

Contact angles of dried membrane droplets cast onto glass slides were measured using a Biolin Scientific Optical Tensiometer (Theta Lite) with OneAttension software. The surface morphologies of as-prepared GO sheets and membranes were characterized by an Olympus BX-51 optical microscope (OM), and a Zeiss EVO SEM. An elemental analysis was investigated using energy dispersive spectroscopy (EDS) to confirm the O/C ratio change of the GO flakes during the plasma treatment. To investigate the topography and surface roughness change of the GO flakes during the plasma treatment, a Veeco Dimension V scanning probe microscope was used in tapping mode using a Veeco OTESPA probe. XPS data was collected using a PHI Quantera Hybrid system using monochromatic Al Kα (1486.6 eV) with a 200 μm X-ray spot size. Infrared spectra of the membranes were taken using a Nicolet iS5 FTIR Spectrometer with iD7 diamond attenuated total reflection (ATR) accessory. The X-ray diffraction data was recorded using a Rigaku SmartLab X-ray diffractometer with Cu Kα (λ = 0.15418 nm) as the X-ray source. The d-spacing of the membranes was calculated by Bragg’s law λ = 2dsinθ, where λ is the wavelength of the X-ray beam, d is the membrane’s interlayer space (d-spacing), and 2θ is the diffraction angle. Thermogravimetric analysis was performed using a TGA 8000 (PerkinElmer). The temperature was ramped from room temperature (RT) to 600 °C at a constant rate of 10 °C/min. The stability of the GO membrane before and after the plasma treatment was estimated by stirring a 1 cm × 1 cm GO membrane in 10 mL of ultrapure water. Images taken during the stirring were collected using a digital camera to assess the extent of membrane disassembly.

Dielectric properties were evaluated using electrochemistry impedance spectra (EIS) (CHI680) in the frequency range 1 Hz–0.1 MHz at room temperature (∼21 °C). GO and PGO membranes were sandwiched between carbon paper electrodes. The direct result from EIS was complex impedance (Z = Zr + i·Zi), which includes both the real part (Zr) and the imaginary part (Zi). The dielectric constant and loss were derived from impedance according to previously established methods.^32–34^

## Results and Discussion

### Plasma Induced Superhydrophilicity

The hydrophilicity of graphene oxide, attributed to the abundance of hydrophilic oxygen-functional groups present on the edges and plane of each GO sheet, also contributes to the aqueous instability of GO membranes. Previous investigations have demonstrated that subjecting the pristine GO membrane surface to oxygen plasma enhances wettability and hydrophilicity, as evidenced by changes in the contact angle.^26,27^ This heightened hydrophilicity is commonly attributed to chemical reactions between the GO material and plasma species, which introduce sp^3^-hybridized groups to the GO surface, generating additional hydrophilic moieties that augment its overall hydrophilicity. As surfaces with water contact angles below 10° are classified as superhydrophilic, we observed superhydrophilicity in GO with just a 10 s exposure to air plasma.

To assess the impact of plasma treatment on hydrophilicity, we measured the contact angle of the treated GO coating (Figure 1a,b). The initial contact angle of the pristine GO coating measured 55 ± 5°, dropping rapidly to below 10° after 10 s or longer plasma treatment. The treated GO coating remains superhydrophilic even with extended plasma treatment duration (Figures 1b and S1). However, the observed superhydrophilicity induced by plasma treatment proved unstable under ambient conditions, with the contact angle gradually increasing over time (Figure 1a,b). Notably, the superhydrophilic state diminished, with the contact angle of the plasma-treated GO coating rebounding to approximately 20° after 2 days and fully recovering to its original value within two months, regardless of the plasma treatment duration (<10 min).

**Figure 1:**
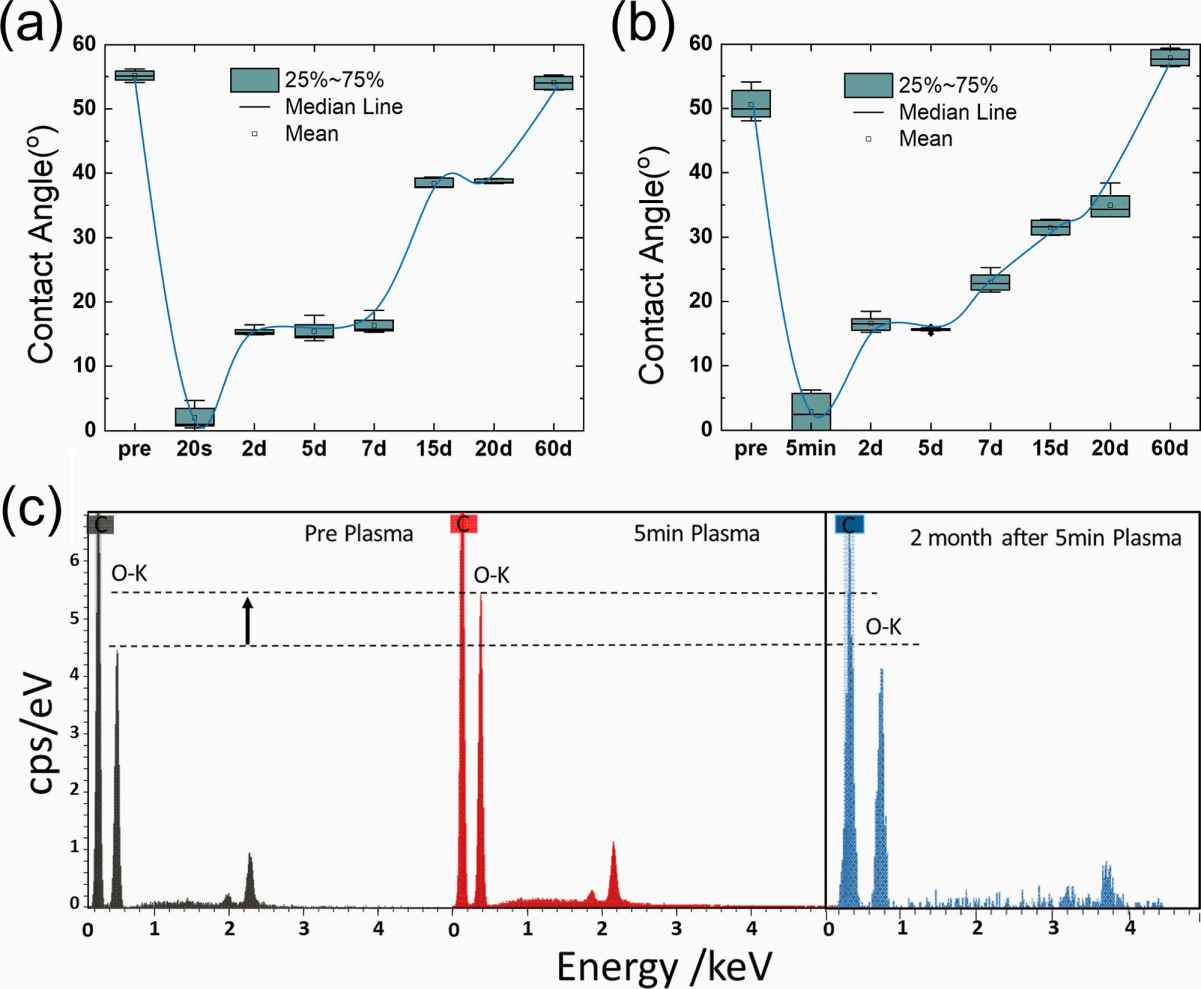
Contact angle changes of GO films before and after plasma treatment for (a) 20 s and (b) 5 min, followed by storage under ambient conditions for up to 60 days. The data are presented as box-and-whisker plots, with a median line connecting the charts to guide the eye. (c) EDS spectrum of one GO membrane (left, black) before plasma treatment, (middle, red) right after 5 min plasma treatment, and (right, blue) left in ambient condition for two months after 10 min plasma treatment.

Based on these wettability observations, we conclude that brief plasma treatment is sufficient to establish a superhydrophilic surface on GO coatings, and extending the treatment duration does not significantly alter the degree of hydrophilicity achieved or the rate of its subsequent recovery (Figure S1). To correlate these wettability changes with surface composition, we conducted energy dispersive X-ray spectroscopy (Figure 1c) on pristine GO, GO treated for 5 min, and aged (two months post-treatment) samples. The EDS spectra demonstrated a change in the oxygen-to-carbon (O/C) ratio upon plasma treatment and subsequent storage. The increased intensity of the oxygen peak (red spectrum) after plasma exposure suggests the introduction of additional OFGs. However, after two months, the oxygen signal diminished to below the pristine level. Despite the insights provided by the EDS analysis, key questions remain unanswered. However, it remains unclear which specific OFGs are formed on the GO surface, as this O/C ratio increase is ambiguous and potentially results from either the introduction of OFGs or the removal of sp^2^-bonded carbon via etching.

### Effect of Surface Modification

To comprehensively analyze the surface chemical transformations of the GO membrane before and after plasma treatment, as well as during ambient storage, XPS spectra were obtained (Figures 2, S2, and Table S1). Figure 2 illustrates the C 1s and O 1s spectra, with the surface atomic content derived from the respective areas under these spectra. The C 1s spectrum of pristine GO reveals graphitic carbon at a binding energy (BE) of 284.6 eV, hydroxyl/epoxy groups (C–O) at 286.8 eV, and carbonyl groups (O–C=O) at 288.7 eV. The O 1s spectrum shows peaks corresponding to C=O and C–O at 531.3 and 532.6 eV, respectively. Table S1 summarizes the surface elemental composition and atomic content for the identified functional groups.

**Figure 2:**
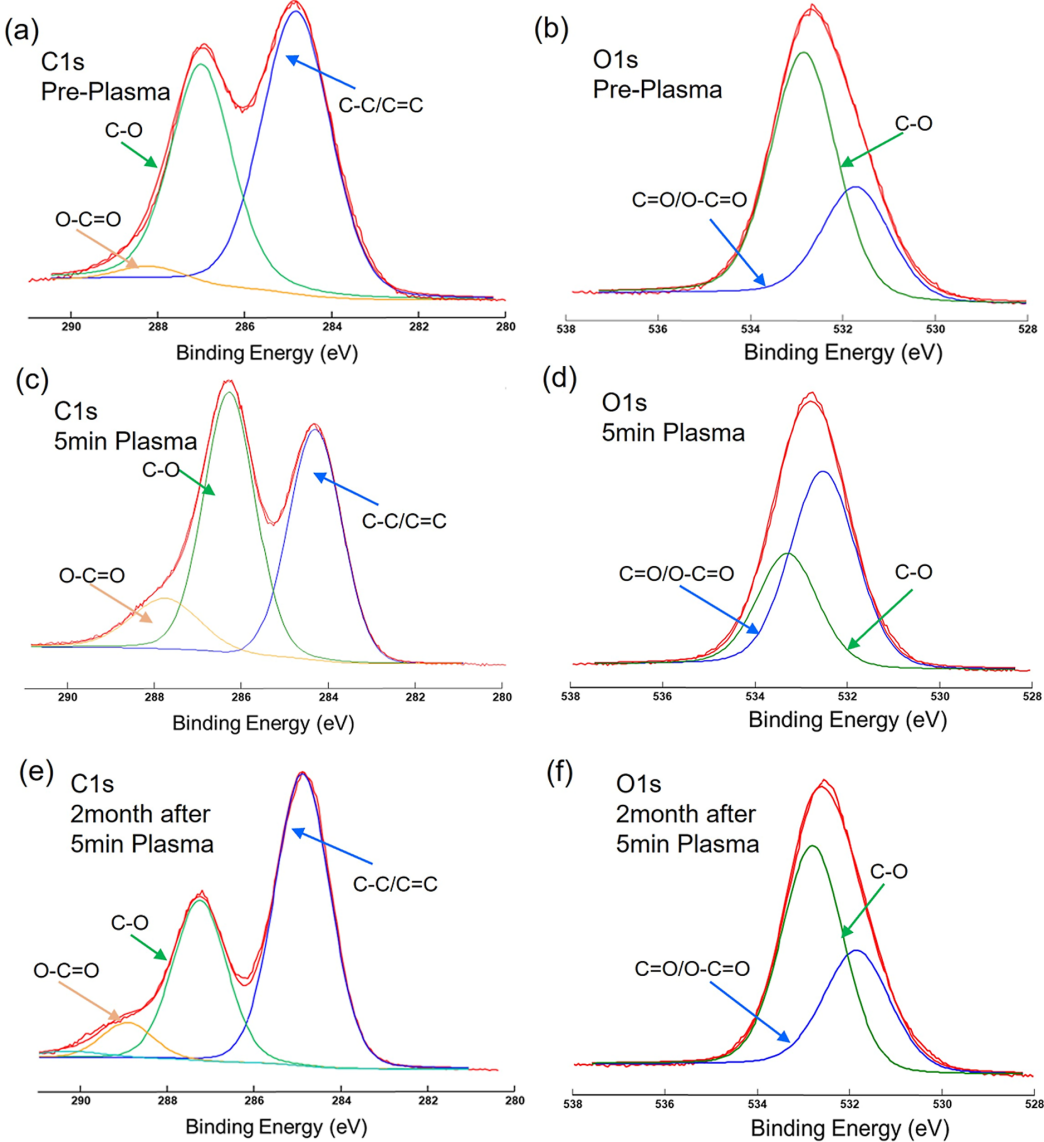
XPS spectra of C 1s (left) and O 1s (right) collected at takeoff angles of 20° for (a,b) pristine GO membrane, (c,d) GO membrane treated with RF air plasma for 5 min, and (e,f) plasma-treated GO membrane after 2 months of storage under ambient conditions.

The XPS analysis provides further insight into the oxidation observed in the EDS results, specifically highlighting the formation and evolution of OFGs during plasma treatment. Examining the C 1s peaks, the intensity of the peak at 284.6 eV (blue, C–C/C=C), corresponding to the graphitic structure, slightly decreases to 25.5% after 5 min of plasma treatment, indicating potential etching within the graphitic framework. In contrast, the intensity of the peak at 286.8 eV (green, C–OH), representing hydroxyl groups, increases from 27.3% to 32.4%, suggesting the formation of hydrophilic functional groups. It is important to note that the atomic concentration of each bond is expressed as a relative percentage, maintaining a constant sum of 100%. After two months of ambient storage, the relative C–O fraction returns to pretreatment levels, suggesting loss of OFGs due to ambient oxidation. The peak at 288.7 eV (yellow, O=C–O), corresponding to carboxyl groups, undergoes a larger relative change from 3.2% to 7.7% after 5 min of plasma treatment, and decreases similarly with subsequent storage.

In the O 1s spectrum, the peak around 532 eV (blue, C=O, carbonyl) increases significantly from 7.4% to 22.4%, while the peak around 533 eV (green, C–OH, hydroxyl) decreases in relative intensity from 23.0% to 12.0% after 5 min of plasma treatment. This shift suggests relatively greater incorporation of carbonyl (C=O) groups relative to hydroxyl (C–OH) groups during plasma treatment. These values show noticeable changes during ambient storage, indicating the ongoing surface modification capability of plasma treatment. In summary, RF air plasma treatment enriches the GO surface with OFGs, resulting in the disruption of sp^2^-bonded carbon. This modification makes the GO surface more susceptible to redox chemistry and loss of OFGs under ambient conditions during storage, as evidenced by both XPS and EDS analyses.

GO is characterized by the presence of various OFGs on its basal plane and edges, which influence its properties such as hydrophilicity and interlayer spacing. These structural and chemical features are further elucidated using techniques like Fourier transform infrared spectroscopy, X-ray diffraction, and thermogravimetric analysis (Figure 3).

**Figure 3:**
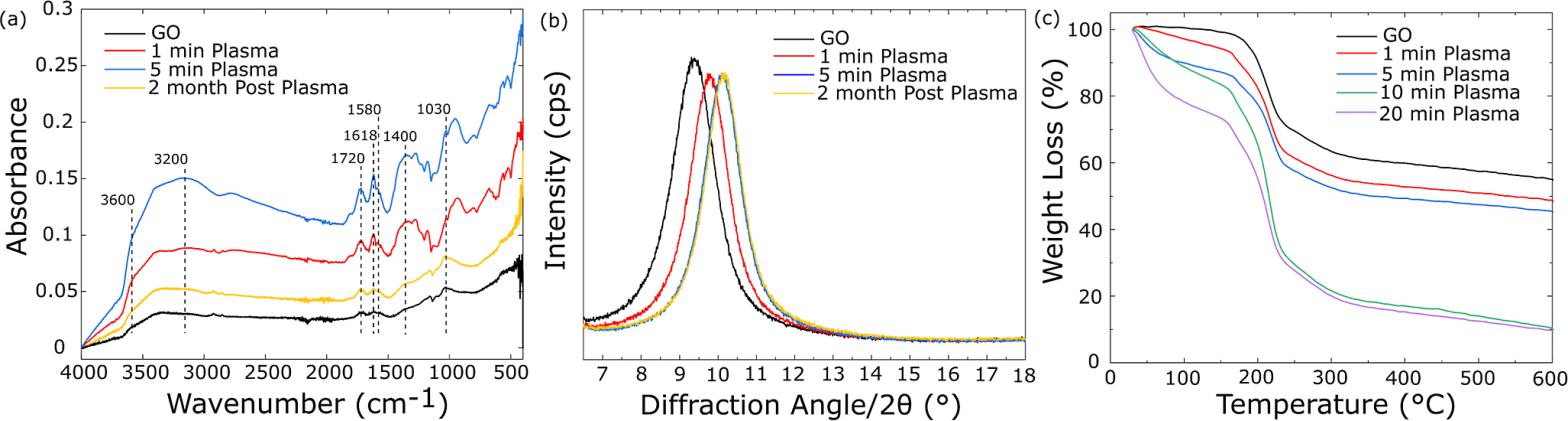
(a,b) FTIR spectrum and XRD pattern of GO, 1 and 5 min plasma-treated GO, and GO stored for 2 months following 5 min plasma treatment. (c) TGA traces of pristine GO and GO are subjected to 1, 5, 10, and 20 min of plasma treatment.

The surface functional groups of the pristine and plasma-treated GO membranes were analyzed using ATR-FTIR. The resulting spectra are presented in Figure 3a. The FTIR spectra of unmodified GO, plasma treated membranes (1 and 5 min), and the GO membrane stored 2 months following 5 min plasma treatment are shown in Figure 3a. Following plasma treatment, significant changes in the spectral features are observed. The water content of the membranes increases as evidenced by growth of the broad, intense feature centered at 3200 cm^–1^ corresponding to water stretching and the sharper peak at 1618 cm^–1^ corresponding to the bending motion of intercalated water.^35–37^ While precise assignments are challenging in the dense fingerprint region of the spectrum, several peaks in regions associated with OFGs increase in intensity, including characteristic bands at 1030 cm^–1^ (C–OH str), 1400 cm^–1^ (br, COH bend), 1720 cm^–1^ (C=O str), and 3600 cm^–1^ (sh, O–H str).^35–37^ In addition, the peak at 1580 cm^–1^ assigned to C=C stretching grows in with plasma treatment. The intensity of C=C stretching depends strongly on the local environment and the observed change in intensity suggests changes to the graphitic surface structure as a result of treatment. A modest increase in the intensity of all features occurs upon increasing plasma treatment time from 1 to 5 min. Following a storage period of 2 months, the FTIR spectrum is nearly indistinguishable from that of the original GO membrane. In agreement with the EDS and XPS data, the FTIR data reveals that significant loss of OFGs occurs under ambient conditions.

X-ray diffraction analysis (Figure 3b) was employed to investigate changes in the interlayer spacing of the laminated GO membranes. The XRD pattern of pristine GO exhibited a characteristic (002) diffraction peak around 10°, corresponding to an initial average interlayer d-spacing.^21,31^ Following plasma treatment, a consistent rightward shift of this peak to 10.4° was observed within the first 5 min, indicating a reduction in the average interlayer distance to approximately 8 nm (calculated using Bragg’s law). The compaction of the GO layers is likely a result of the etching process. While the removal of intercalated water molecules may also contribute by decreasing interlayer spacing, the extent to which this water is readsorbed under ambient conditions remains unclear. Hydrophilicity measurements, XPS, and FTIR data collectively demonstrate significant surface modification of the GO membranes following plasma treatment. However, while XRD results indicate a change in the interlayer spacing of the GO layers, suggesting a potential alteration in the material’s bulk structure, this change could also be influenced by the removal of intercalated water, as discussed earlier. Therefore, the XRD data primarily suggests changes in the GO interlayer spacing, with the precise nature of bulk structural changes requiring further thermal analysis.

To further investigate the potential for bulk structural changes, the thermal behavior of pristine and plasma treated GO membranes was investigated using TGA under nitrogen atmosphere from room temperature to 600 °C (Figure 3c). The TGA profiles displayed three characteristic weight loss stages. The initial weight loss (Region I, up to 100 °C) due to water evaporation was significantly more pronounced in PGO samples, with the extent of loss increasing with plasma treatment time, consistent with the FTIR data indicating enhanced water adsorption. In the second stage (Region II, 100–200 °C), PGO membranes exhibited a greater degree of decomposition compared to pristine GO, and this weight loss also increased with plasma duration. This observation suggests an increased population of OFGs, such as carboxyl, epoxy, and lactone, introduced by the plasma treatment, which are less thermally stable. This finding reinforces the results from XPS and FTIR, which identified the introduction of hydrophilic OFGs due to plasma treatment. The increase in OFGs aligns with the observed increase in hydrophilicity (reduced contact angle), as these polar functional groups enhance the interaction with water. The final decomposition stage (Region III, 200–600 °C), corresponding to the breakdown of the carbon skeleton and more stable carbonyl/quinone groups, showed comparable weight loss percentages across all samples. These results suggest that while plasma treatment induces significant changes in surface functionality, it appears to have a relatively minor effect on the thermal stability associated with the GO framework.

### Etching and Exfoliation

The decline in the sp^2^-hybridized carbon atomic concentration, as revealed in the XPS analysis, suggests the etching of graphene oxide. The physical collision of the high-energy plasma particles onto the surface of GO sheets can directly bombard sp^2^ bonded carbon atoms and decorated OFGs, which are expected to change the surface roughness. To gain insights into and quantify the etching of GO under RF air plasma, we employed a multifaceted approach to characterize the morphology of both the GO membrane and coating by utilizing optical microscopy and scanning electron microscopy (Figures 4 and S3). During a 10 min plasma treatment, a noticeable increase in transparency of the GO coating (2 mL of 0.1 mg/mL GO solution, coating area of 2 cm × 5 cm) was discernible to the naked eye, as shown in Figure 4a. Upon extending the plasma treatment time to 30 min, the thinner regions of the GO coating almost entirely underwent etching. Figure 4b displays corresponding optical microscope images of the GO coating at the same time points. The blue circles highlight specific wrinkles or folded regions of the GO film. Notably, despite the increasing transparency observed in (a), the morphology and location of these circled wrinkles appear to remain consistent throughout the 30 min treatment. The consistent morphology of the circled wrinkles in Figure 4b, even as the overall transparency increases, strongly suggests that the etching process is primarily vertical, removing GO material uniformly from the top surface without significantly altering the underlying folded structures. This indicates an anisotropic etching behavior, proceeding downward from the exposed surface. Figure 4c,d present SEM cross-sectional images of the GO coating before (0 min) and after 20 min of plasma treatment, respectively. The initial thickness of the GO coating is approximately ∼ 2.0 μm (Figure 4c). After 20 min of plasma treatment, the thickness appears to increase to ∼ 2.2 μm (Figure 4d). This observation of a relatively small change in cross-sectional thickness, even with a significant increase in transparency, further supports the idea of gentle exfoliation. The observed increase in transparency (Figure 4a) during the initial 10 min of plasma treatment, while SEM images over larger areas (Figure S3) and AFM roughness measurements showed no significant alterations, likely indicates a subtle thinning of the GO layers occurring at a nanoscale level or a change in the refractive index of the material as OFGs are removed. These changes might not be readily apparent as large-scale morphological changes or significant roughness variations within the initial stages. The consistent presence and morphology of the wrinkles observed under the optical microscope throughout the etching process strongly suggest that these features were formed during the initial deposition or drying of the GO coating and are inherent to the film’s structure from the bottom up, rather than being newly formed or significantly altered by the plasma treatment.

**Figure 4:**
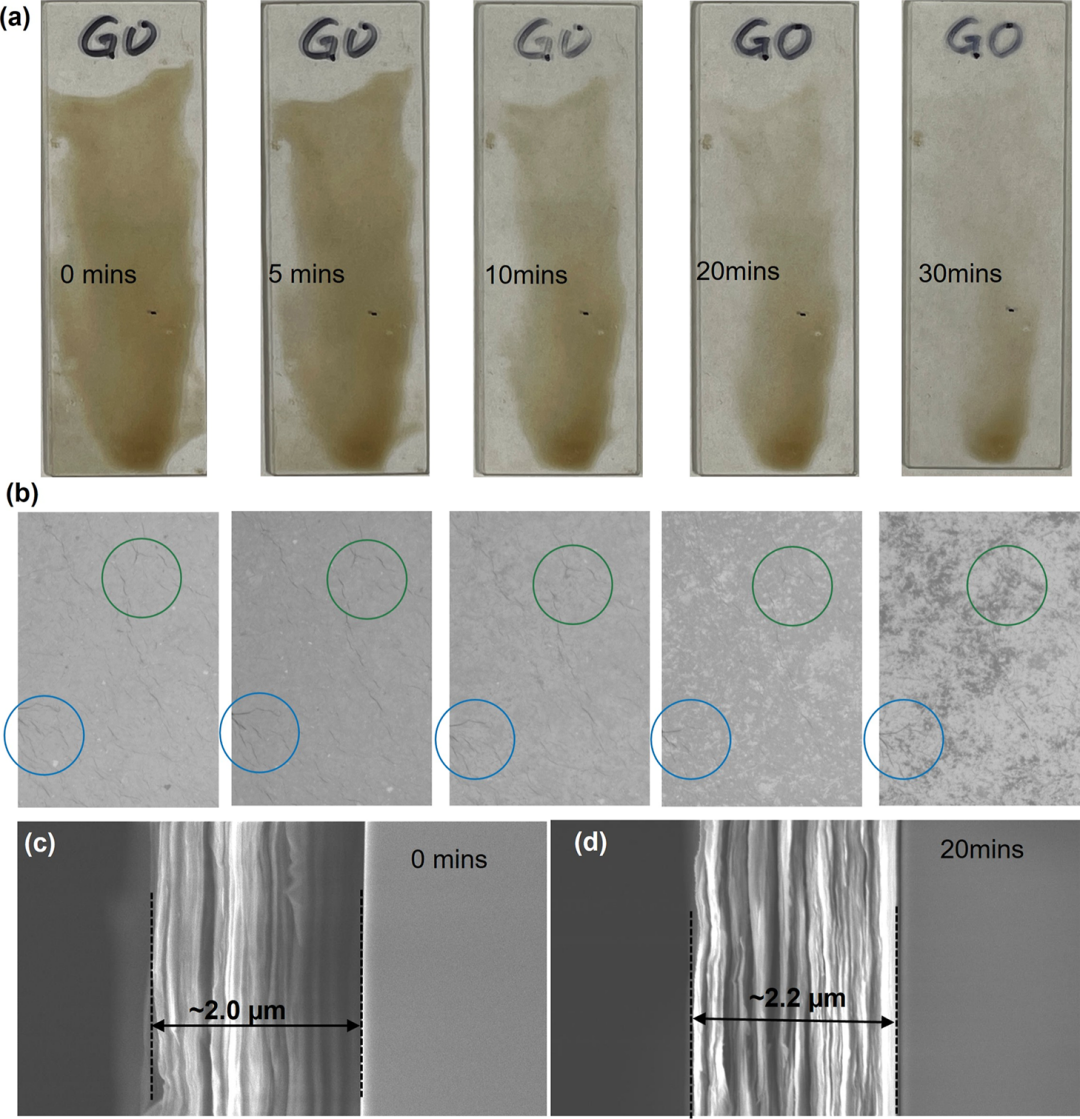
Effect of plasma treatment on GO coating morphology: (a) camera images and (b) matching optical microscope images of a GO coating on glass slides at increasing plasma treatment times (0–30 min). SEM cross-sectional images of the GO coating on a Si wafer (c) before and (d) after plasma treatment (20 min).

This anticipates changes in surface profilometry as long as we can decrease the thickness of the GO sample. To investigate the plasma induced etching of GO, individual GO sheets were characterized using atomic force microscopy (middle column) and compared with their appearance under an optical microscope (left column) after plasma-treatment, as illustrated in Figure 5. Figure 5 presents the surface morphology (optical: 500 μm × 500 μm scale, AFM images vary in size) of an individual GO sheet, depicting its evolution with each additional minute of plasma treatment, from 0 min (a) to 4 min (e), until the sheet becomes nearly invisible under the optical microscope (e). The observations reveal both a visual decrease in the size and contrast of the GO sheets under the optical microscope (left column, (a) to (e), scale bar 10 μm), indicative of thinning, and the development of irregularities and etching pits visible in the AFM topography images (middle column, (b) onward) as plasma treatment time increases. After 2 min of plasma treatment (c), the GO sheet exhibits increased porosity and significantly diminished visibility under the optical microscope, with faint GO sheet residue potentially remaining on the wafer surface as suggested by the AFM image. A detailed analysis of the height (thickness) of the GO sheets, presented in the height profiles (right column) corresponding to the white lines in the AFM images, indicates a consistent height decrease with increasing plasma treatment time (Figure S4). Concurrently, analysis of the average surface roughness (Ra) (Figure S4) reveals an initial rapid decrease from 25 nm for the pristine sample to 2–3 nm after 1 min of plasma treatment (Figure 5b). While visual inspection of the AFM images (middle column, Figure 5) clearly shows the progressive development of etching pits and irregularities with extended treatment time, the average roughness (Ra) largely stabilizes, with no significant additional impact observed even after 4 min of plasma treatment (Figure 5e). This apparent paradox can be reconciled by considering the nature of plasma etching at the atomic level and the statistical definition of Ra. The uniform removal of material across the GO surface, combined with an increase in defect density and localized grain refinement, leads to an overall reduction in the average height deviation (Ra), particularly by smoothing out larger pre-existing irregularities on the pristine GO. Although individual pits form, their uniform distribution and atomic-scale depth may not significantly increase the overall Ra value, which is an average metric. Instead, these processes collectively create a more refined surface morphology, characterized by a decrease in overall surface roughness alongside the development of distinct etching features.

**Figure 5:**
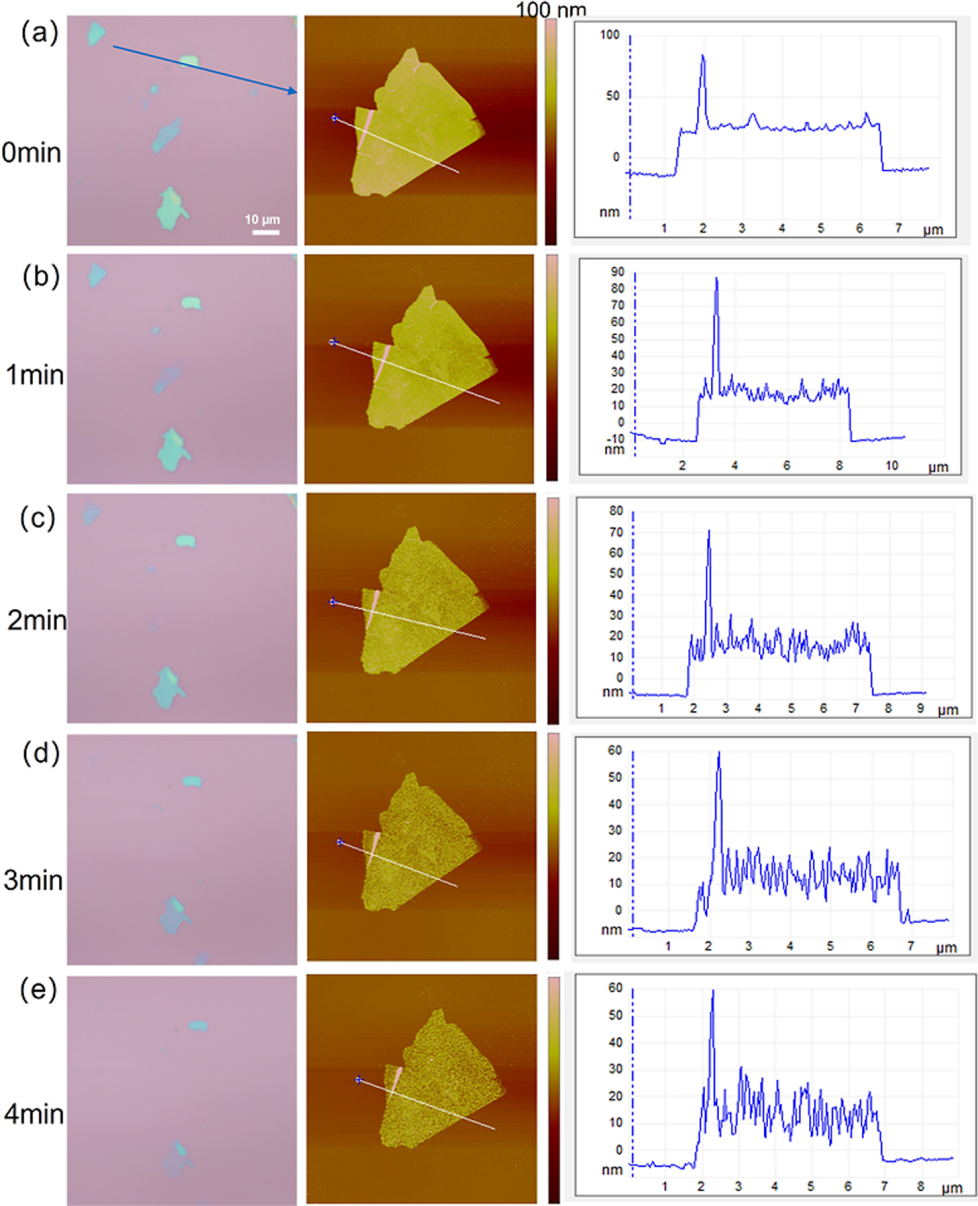
Monitoring plasma etching of a graphene oxide sheet using optical microscopy (left), AFM (middle), and height profiles (right) at different treatment times: (a) 0 min, (b) 1 min, (c) 2 min, (d) 3 min, and (e) 4 min. The height profiles represent cross sections of the AFM topography. To discern and quantify the impact of plasma etching on the GO surface, individual GO sheets were subjected to plasma treatment, diverging from the typical approach involving laminated GO coatings or membranes. The air plasma environment is known to comprise fast electrons, ions, and neutral species that impinge upon the sample surface, potentially altering its surface roughness.

### Dielectric Performance Improvement

As the dielectric properties of GO are highly dependent on the OFGs decorated on the GO sheet and the resulting lamination of these GO sheets, any modification affecting the OFGs and the GO sheet stacking will consequently impact the dielectric properties of the GO membrane. Previous research has shown that cation modification can improve the aqueous stability of GO membranes and tends to decrease their dielectric constant due to strengthened inter/intra sheet bonding and more organized stacking.^32–34^ In this part, we investigate the dielectric constant (DiC) and dielectric loss (DiL) of both pristine GO and PGO membranes by forming planar dielectric capacitors, where the GO/PGO membrane is sandwiched between carbon paper electrodes. As stated above, plasma treatment is expected to infuse the GO surface with OFGs and disrupt the lamination through oxidation, leading to gentle exfoliation. Therefore, a positive impact on the dielectric performance of GO membranes postplasma treatment was anticipated. Frequency-dependent DiC and DiL of GO and PGO membranes were examined across a range from 0.01 Hz to 1 MHz (Figure 6), and the parameters were estimated based on impedance data (Figure S5). Figure 6a shows the frequency dependence of the dielectric constant, while Figure 6b illustrates the frequency dependence of the dielectric loss for pristine GO (gray line) and GO membranes treated with RF air plasma for varying durations (red: 1 min, blue: 2 min, green: 5 min, purple: 10 min, yellow: 15 min). Our results, visualized in Figure 6, indicate that a short plasma treatment of 1 min (red line) has a minimal impact on both the dielectric constant and dielectric loss across the measured frequency range, as the curves largely overlap with the pristine GO (gray line). Instead, with increasing plasma treatment time (blue, green, purple, and yellow lines in Figure 6a), a clear increase in the dielectric constant is observed specifically at lower frequencies (<100 Hz). Conversely, Figure 6b shows a noticeable decrease in dielectric loss in the medium frequency range (0.1–10 kHz) for samples treated with longer plasma durations compared to the pristine GO membrane. Despite the expected increase in DiC due to the enrichment of OFGs and the creation of vacancy defects through etching, the impedance drop at low frequency with plasma time increase is observed in Figure S5 contradicts this expectation. Potential explanations for this discrepancy include surface oxidation, intersheet water loss, and the electrode/dielectric layer interface improvement postplasma treatment. It is important to note that the observed improvements in dielectric properties postplasma treatment may be susceptible to environmental factors. Preliminary observations suggest that these enhancements can diminish over time when the membranes are exposed to ambient air in nonsealed environments such as regular zip bags or Petri dishes. Conversely, maintaining the treated membranes in a sealed environment, like a glovebox, appears to preserve these enhanced dielectric characteristics.

**Figure 6:**
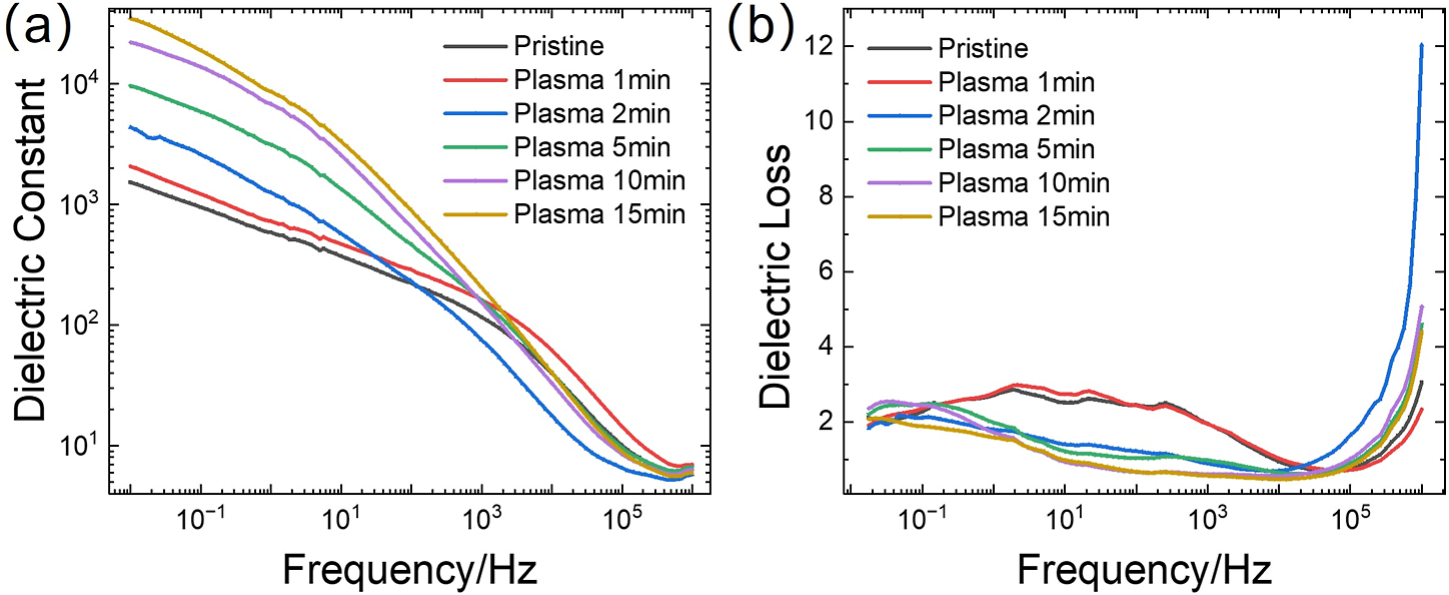
Frequency dependence of dielectric constant (a) and dielectric loss (b) for pristine GO and plasma-treated GO membranes.

Based on the high dielectric constant of GO materials, one of the critical applications of GO membranes as dielectric materials lies in their use as the dielectric substrate for electronics and separator membranes in capacitors. The electrochemical performance of the GO/PGO as a dielectric capacitor can be estimated through typical electrochemical cell testing. To ensure data comparability, the same pair of carbon paper electrodes was employed in securing the cyclic voltammetry (CV) analysis of sandwich-structured carbon paper-GO/PGO-based membrane-carbon paper (C-GO/PGO-C) capacitors, utilizing either GO or PGO membranes as dielectric separators. CV analysis offers insight into the capacitive performance of these devices. The CV curves were obtained at a scan rate of 100 mV/s within the potential window of −1 to 1 V. Figure 7a shows that typical CV curves are nearly rectangular, indicating an efficient double-layer formation and high performance. As shown in Figure 7a, the area of the CV curve increases with increasing plasma treatment time, and the peak current value generally increases with plasma treatment time up to 10 min. A comparison of peak current (black squares) and charge/discharge current difference at 0 V (red triangles) before (pristine) and after different plasma treatments at the scan rate of 100 mV/s was performed, as shown in Figure 7b. The linear increase in peak current and the charge/discharge current difference at 0 V with increasing plasma treatment time (Figure 7b) suggest an enhanced charge storage capability and improved capacitive behavior of the PGO-based capacitors. Figures 7c and S6 confirm that such improvement does not change with the scan rate. It was observed that the capacitor fabricated with GO exposed to plasma for 15 min (yellow line in Figure 7a) shows a significantly larger area compared to the untreated ones, likely due to interface improvement. Such improvement is stable over a long-time range as the dielectric layer is not exposed to air and moisture. Figure 7d presents a Ragone plot, illustrating the specific power and specific energy for the PGO capacitors. The arrow indicates the general trend of increasing specific power and specific energy with longer plasma treatment times, suggesting improved overall energy storage performance. The deviation from ideal rectangular CV curves and linearity in hypothetical charge/discharge behavior is likely caused by a combination of electric double-layer capacitance (EDLC) from the graphene sheets and pseudocapacitance from residual oxygen groups. This facile plasma treatment strategy enhances the electrode/electrolyte interface, resulting in improved CV curve rectangularity, and increased specific power and energy. Such improvement is stable during the storage as long as the dielectric layer is not exposed to air and moisture (Figure S7). The exfoliation of GO membranes caused by the extended plasma treatment, combined with the infusion of OFGs, could be responsible for the DiC increase and DiL decrease, potentially leading to a better electrode/dielectric interface (e.g., improved wettability and contact with the carbon paper). Our previous studies suggest that higher dielectric performance correlates with more disordered GO stacking.^32–34^ Therefore, a decrease in aqueous stability after plasma treatment is expected.

**Figure 7:**
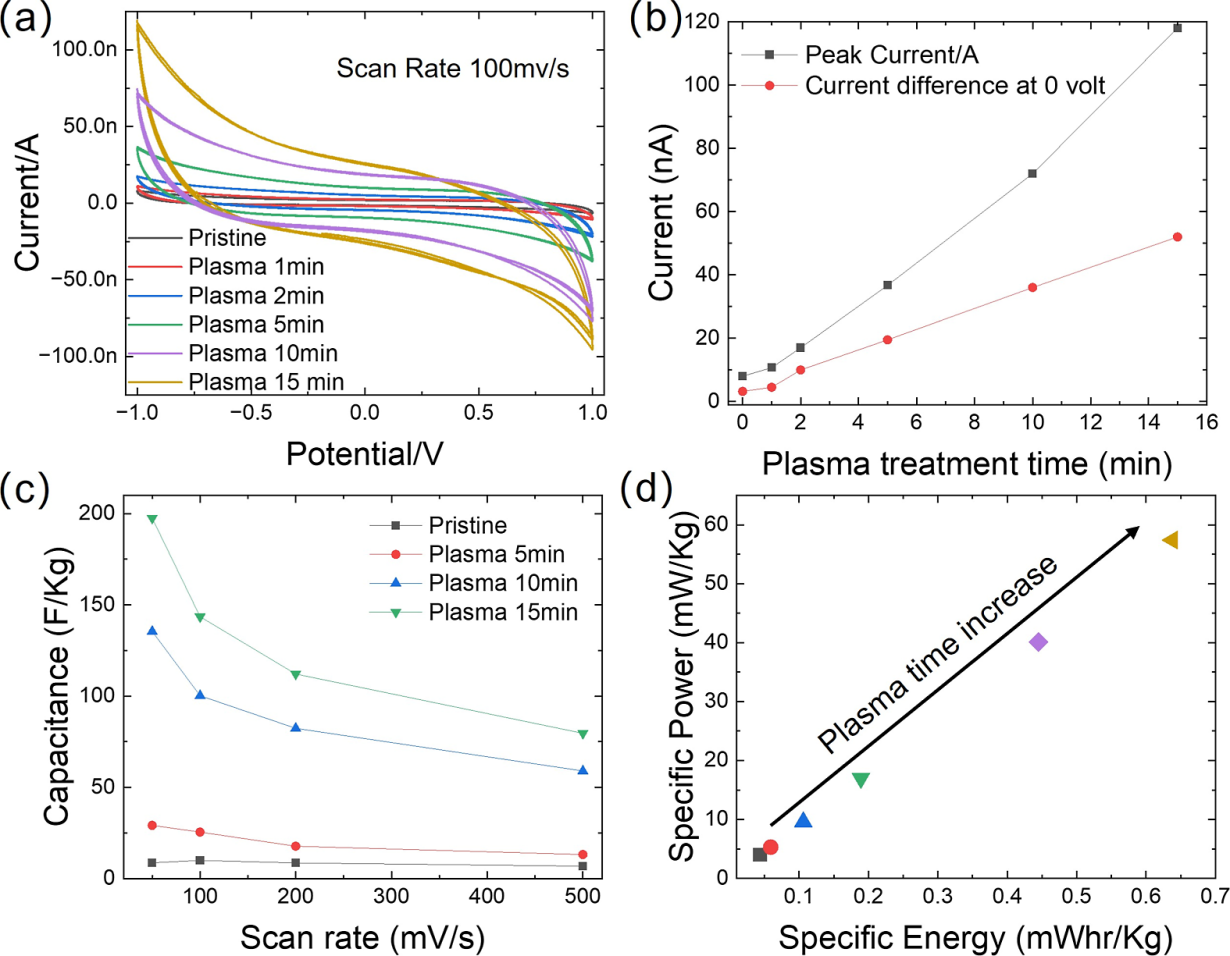
Capacitive performance of carbon paper capacitors with GO/PGO dielectric separators. (a) Cyclic voltammetry curves at 100 mV/s (−1 to 1 V) for pristine GO and PGO with different treatment times; (b) Peak current and current difference at −1 V from (a) plotted against plasma treatment time; (c) Specific capacitance vs scan rate; (d) Ragone plot illustrating the specific power and specific energy of PGO capacitors with increasing plasma treatment time.

### Aqueous Stability Decrease

The aqueous stability of GO membranes, a crucial factor for their applications in aqueous environments, is significantly compromised following plasma treatment. While pristine GO membranes exhibit inherent instability in water due to electrostatic repulsions and hydrophilic OFGs, plasma treatment further exacerbates this issue by inducing superhydrophilicity through surface modification and etching, leading to the formation of a more porous structure. Plasma treatment cleaves covalent bonds, generating free radicals that rapidly form chemically activated functional groups such as—COOH, carbonyl moieties, and—OH. These newly formed groups significantly enhance the hydrophilicity of the GO membrane, leading to immediate and complete wetting upon water contact. Experimental observations reveal that under gentle stirring at 200 rpm (Figure 8), GO membranes begin to disintegrate within 20 min, with complete disassembly occurring after 30 min. In stark contrast, plasma-treated GO membranes, exposed to plasma on both sides for just 1 min, disintegrate entirely within 10 min of stirring. This rapid disintegration is attributed to the immediate and complete wetting of the superhydrophilic GO surface, which facilitates water penetration between the GO layers, weakening the intersheet interactions. The increased surface area due to porosity also contributes to this instability. The disintegration process involves rapid surface wetting, followed by the detachment of individual GO sheets due to the shearing forces of stirring, which are now able to overcome the weakened cohesive forces. Furthermore, the GO membrane’s surface layer, potentially offering some initial protection, is compromised by plasma treatment through the introduction of additional hydrophilic groups and defects. The observed increase in hydrophilicity postplasma treatment, driven by the rapid formation of C–O, C=O, COOH, and—OH bonds, directly correlates with the diminished aqueous stability, highlighting a critical trade-off between enhanced surface functionality and structural integrity in aqueous environments.

**Figure 8:**
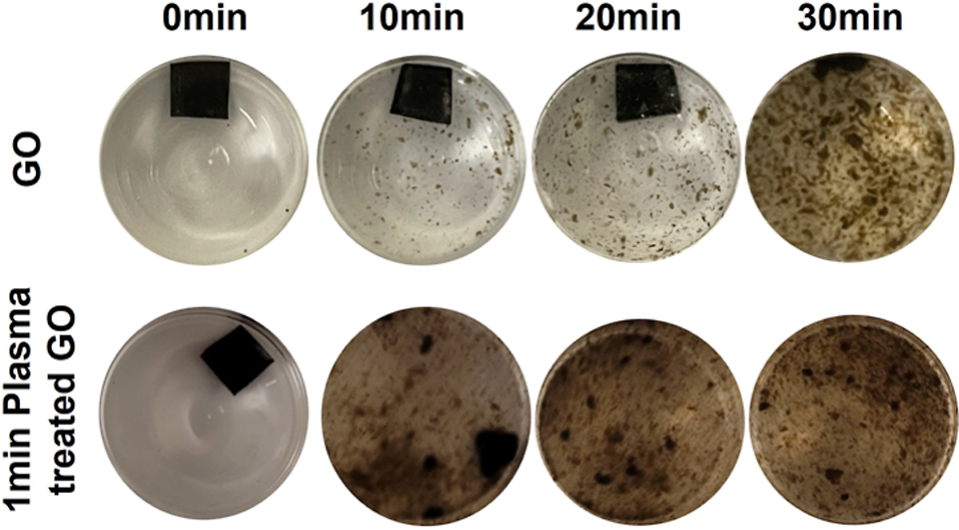
Stability of pristine and plasma-treated GO membranes in ultrapure water under stirring at 200 rpm.

## Conclusion

This study comprehensively characterized the surface modifications induced by RF air plasma treatment on GO films, revealing a nuanced interplay between oxygen functionalization and etching. Contact angle measurements demonstrated a rapid transition to superhydrophilicity, correlating with increased oxygen content and specifically carbonyl groups detected by EDS and XPS. AFM revealed atomic-level etching, while XRD indicated a denser stacking of GO sheets. These structural and chemical alterations enhanced the GO-carbon paper electrode interface, as evidenced by improved performance in cyclic voltammetry and electrochemical impedance spectroscopy, suggesting promise for dielectric applications, albeit with a noted reduction in aqueous stability. Importantly, optical microscopy confirmed that the GO film maintained its microlevel surface roughness and wrinkle integrity until the plasma reached the bottom layers of the coating. In essence, RF air plasma treatment effectively tailors GO surface properties, optimizing them for dielectric applications through controlled oxidation and structural rearrangement. However, precise control of plasma exposure duration is crucial, as extended treatment induces excessive porosity and oxidation, limiting suitability for aqueous environments. Consequently, this research underscores the necessity of meticulously tailoring plasma treatment parameters to specific application requirements to balance enhanced functionality with desired stability.

## Supplementary Material



## Notes

Surface atomic concentration; contact angle of GO membranes following 1- and 10 min plasma treatments; SEM surface images; XPS full spectra; average height and roughness; Nyquist and Bode plots of GO/PGO membranes; and cyclic voltammetry curves of carbon paper capacitors with GO/PGO dielectric separators (PDF)
