# Supplementary material for: Plasma-Induced Tailoring of Graphene Oxide Surfaces for Electrochemical Applications: Functionalization and Etching
Source: ACS Appl Electron Mater. 2025 Jun 29;7(14):6635–45. doi: 10.1021/acsaelm.5c00939 (PMC12288062; doi:10.1021/acsaelm.5c00939)
Supplement: Supplementary file 1 [file el5c00939_si_001.pdf]

Supporting Information for

Plasma-Induced Tailoring of Graphene Oxide Surfaces for Electrochemical Applications: Functionalization and Etching

Yijing Y. Stehle,[1]* Timothy J. Barnum,[2] Sandra Schujman,[3] Ivan V. Vlassiouk[4], and Rebecca Cortez[1]

[1] Department of Mechanical Engineering, Union College, Schenectady, NY 12308

[2] Department of Chemistry, Union College, Schenectady, NY 12308

[3] NY CREATES, Albany, NY 12203

[4] Center for Nanophase Materials Sciences, Oak Ridge National Laboratory, Oak Ridge, TN, 37831

* To whom correspondence may be addressed. Email: stehley@union.edu

Table of contents

Table S1. Surface atomic concentrations determined from X-ray photoelectron spectroscopy spectra of graphene oxide (GO) and plasma-treated graphene oxide (PGO) membranes. **3**

Figure S1. Contact angle measurements of PGO films during ambient storage. **4**

Figure S2. X-ray photoelectron spectroscopy full spectra of GO. **5**

Figure S3. Scanning electron microscopy surface images of GO/PGO membranes. **6**

Figure S4. Average height and average roughness of GO sheets as a function of plasma treatment time. **7**

Figure S5. Nyquist and Bode plots of carbon paper capacitors with GO/PGO dielectric separators at different plasma treatment time. **8**

Figure S6. Cyclic voltammetry curves of carbon paper capacitors using GO/PGO as dielectric separators with different scan rate. **9**

Figure S7. Cyclic voltammetry curves of carbon paper capacitors during storage. **10**

Table S1. Surface atomic concentrations (at. %) determined from X-ray photoelectron spectroscopy spectra of graphene oxide (GO) and plasma-treated graphene oxide (PGO) membranes.

GO membrane	C^{1s}(C-C)	C^{1s}(O-C)	C^{1s}(O-C=O)	O^{1s}(C=O)	O^{1s}(C-O)
Pre-plasma	39.04	27.34	3.17	7.42	23.04
5 min plasma	25.50	32.41	7.72	22.40	11.97
2 months later	35.59	27.93	4.95	12.58	18.47

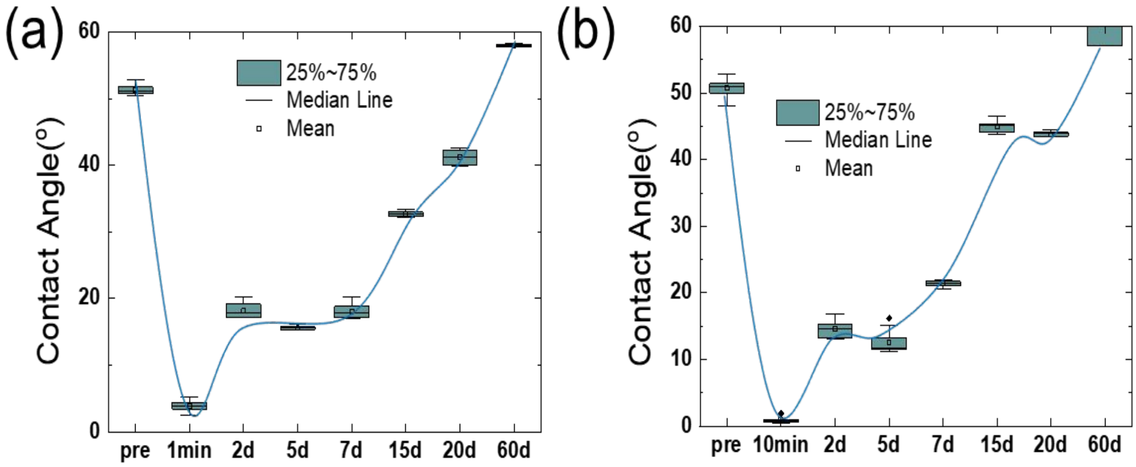

Figure S1. Contact angle measurements of GO films before and after 1-minute (a) and 10-minute (b) plasma treatment, and during storage under ambient conditions for up to 60 days.

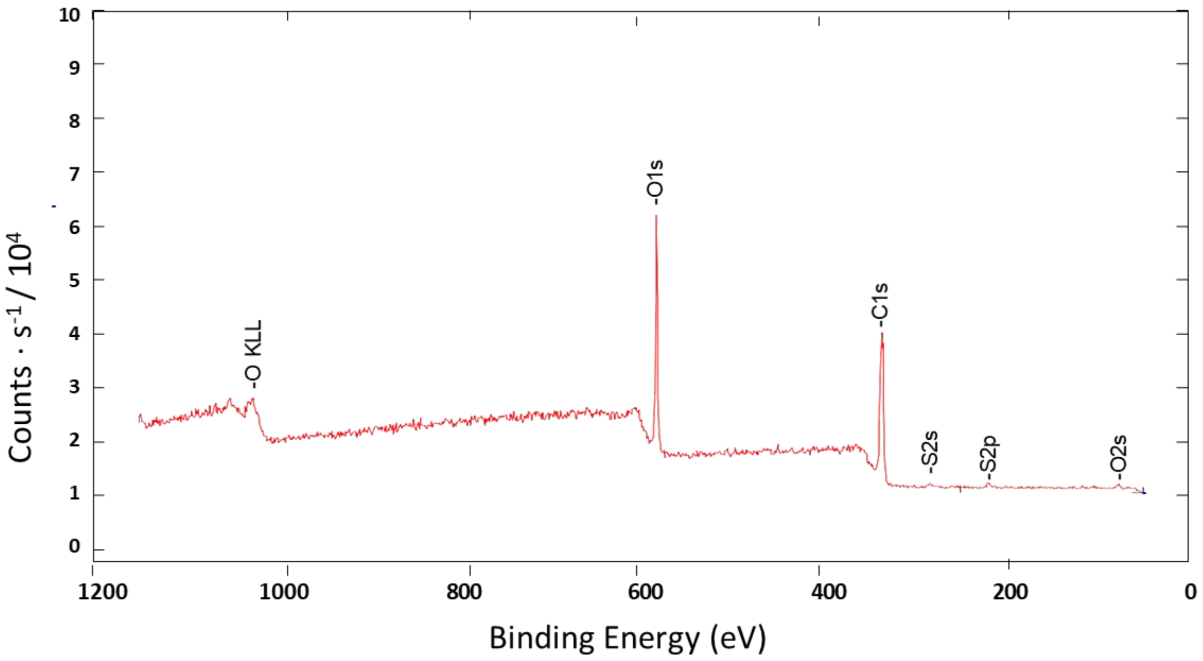

Figure S2. X-ray photoelectron spectroscopy full spectra of GO. No discernible nitrogen peak was observed following plasma treatment.

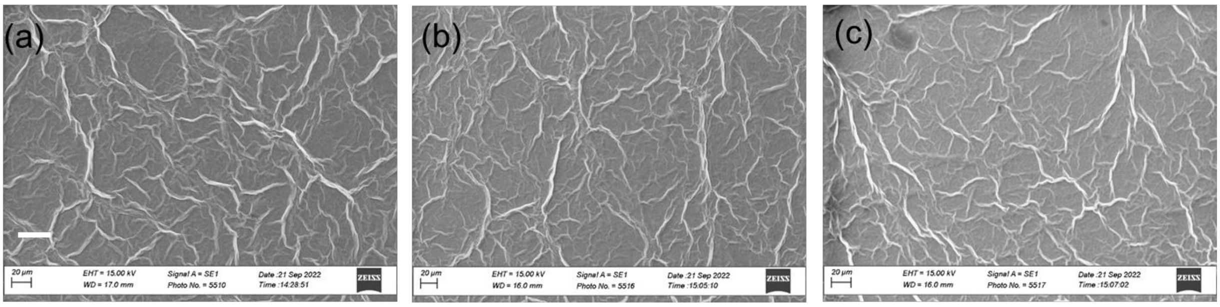

Figure S3. Scanning electron microscopy surface images of (a) pristine GO, (b) 1-minute PGO, and (c) 5-minute PGO membranes. No significant surface morphology differences were observed.

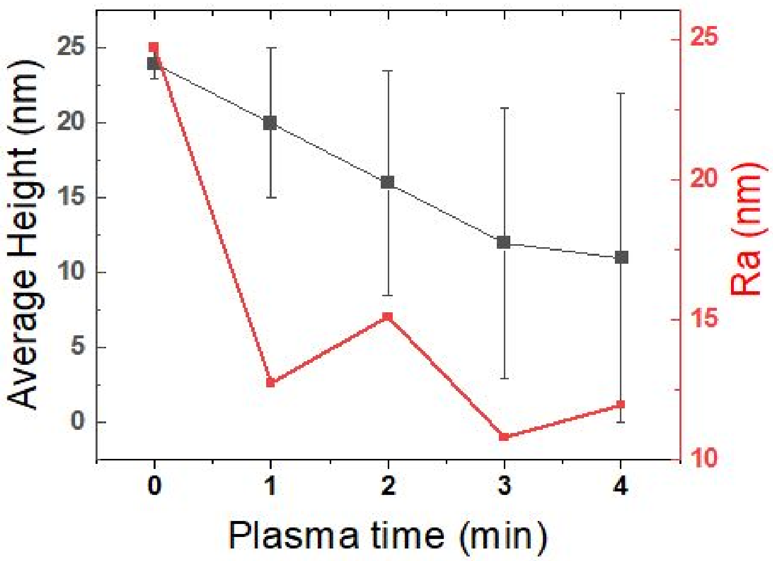

Figure S4. Average height (black squares, left axis) and average roughness (Ra, red line, right axis) of GO sheets as a function of plasma treatment time (minutes). Error bars represent standard deviations.

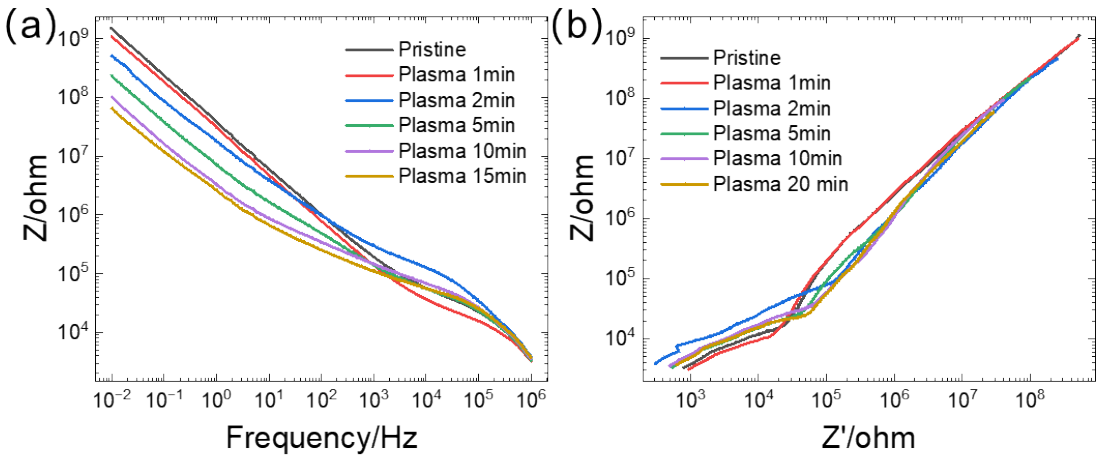

Figure S5. Nyquist (a) and Bode (b) plots demonstrating the change in impedance characteristics of carbon paper capacitors with GO/PGO dielectric separators as a function of plasma treatment time (1 to 20 minutes).

The frequency dependence of the dielectric constant (DiC) and dielectric loss (DiL) of GO membranes in the frequency range from 1 Hz to 1 MHz are estimated based on impedance data (Fig. S5) using equation (S1), (S2), and (S3),

$$\varepsilon = \varepsilon' - i\varepsilon'' = \frac{d \cdot Z''}{A \cdot 2\pi f \cdot |Z|^2} - i\frac{d \cdot Z'}{A \cdot 2\pi f \cdot |Z|^2} \qquad \text{(S1)}$$

$$\text{DiC} = \varepsilon_r = \frac{\varepsilon'}{\varepsilon_0} = \frac{d \cdot (-Z'')}{A \cdot 2\pi f \cdot |Z|^2 \cdot \varepsilon_0} \qquad \text{(S2)}$$

$$\text{DiL} \approx \tan\delta = \frac{\varepsilon''}{\varepsilon'} = \frac{-Z'}{Z''} \qquad \text{(S3)}$$

where ε is the complex permittivity ($F \cdot m^{-1}$), Z is the complex impedance (Ohm), f is the frequency (Hz), A represents the metal foil area (m^2), d is the separation between the plates in meters, ε_r is the relative permittivity or DiC, and ε_0 is the vacuum electric permittivity (ε_0 $\approx 8.854 \times 10^{-12}$ $F \cdot m^{-1}$).

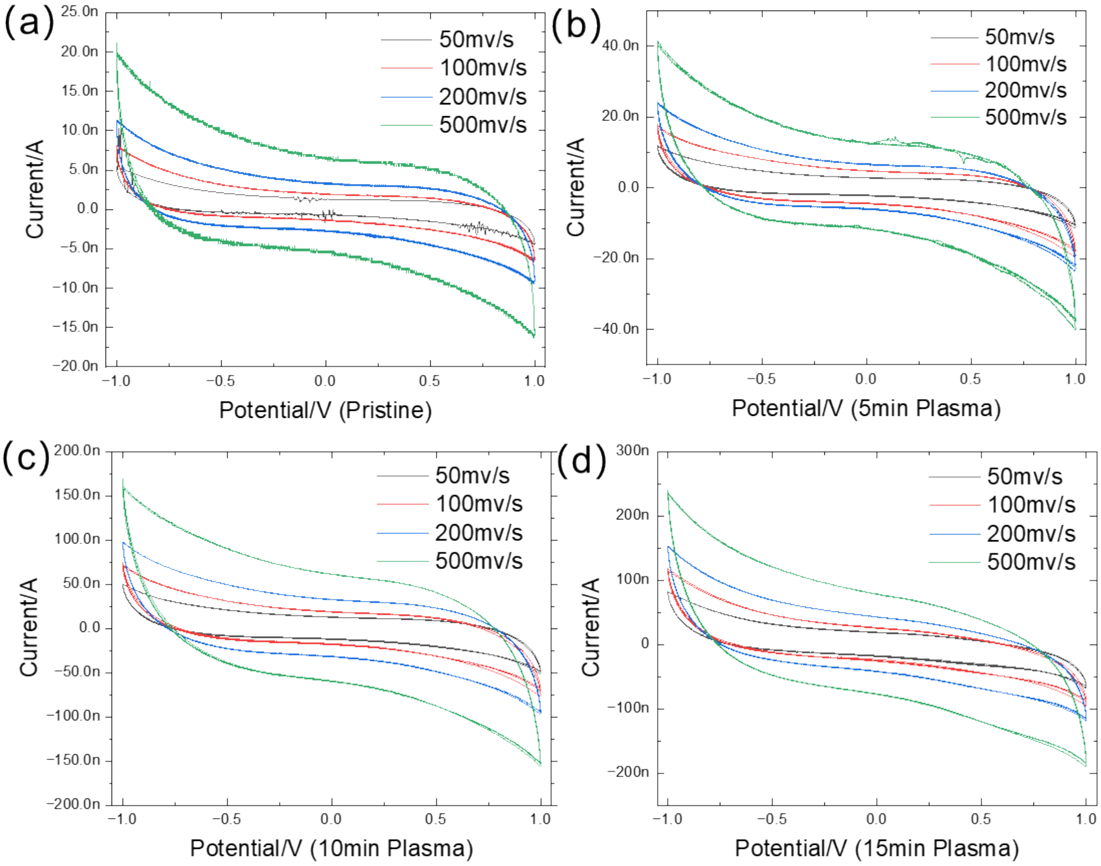

Figure S6. Cyclic voltammetry curves obtained at varying scan rates (±1 V potential window) for carbon paper capacitors employing different dielectric separators, illustrating the impact of plasma treatment time on electrochemical performance: (a) pristine GO, (b) 5-minute plasma-treated PGO, (c) 10-minute PGO, and (d) 15-minute PGO.

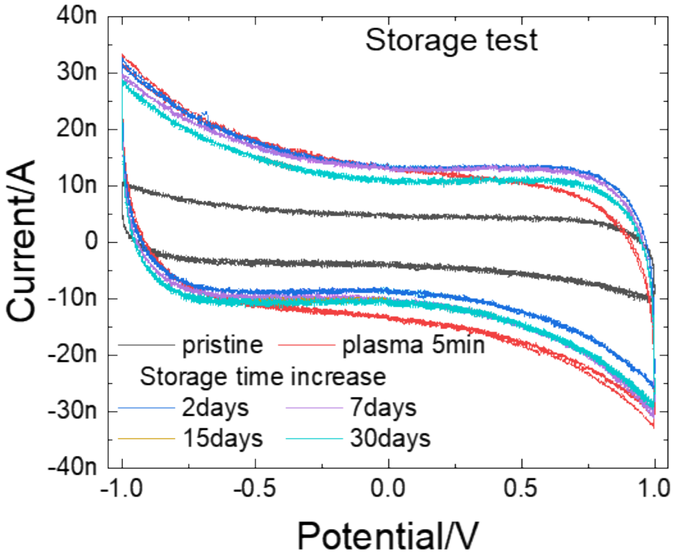

Figure S7. Cyclic voltammetry curves of carbon paper capacitors, using 5-minute plasma-treated PGO as dielectric separators, showing performance stability over one month of storage under air and moisture free condition. Measurements were taken at a scan rate of 100 mV/s within a potential window of ±1 V.
